# Reliability and Remaining Life Assessment of an Electronic Fuze Using Accelerated Life Testing

**DOI:** 10.3390/mi11030272

**Published:** 2020-03-06

**Authors:** Noor Muhammad, Zhigeng Fang, Syed Yaseen Shah, Daniyal Haider

**Affiliations:** 1 College of Economics and Management, Nanjing University of Aeronautics & Astronautics, Nanjing 210016, China; Zhigengfang@163.com; 2 School of Computing, Engineering and Built Environment, Glasgow Caledonian University, Glasgow G4 0BA, UK; syedyaseen.shah@gcu.ac.uk; 3 Department of Electrical and Electronics Engineering, University of Surrey, Guildford GU2 7XH, UK; daniyalhaider86@gmail.com

**Keywords:** one short system, electronic fuze, reliability, remaining useful life (RUL), accelerated life testing (ALT)

## Abstract

An electronic fuze is a one-shot system that has a long storage life and high mission criticality. Fuzes are designed, developed, and tested for high reliability (over 99%) with a confidence level of more than 95%. The electronic circuit of a fuze is embedded in the fuze assembly, and thus is not visible. Go/NoGo fuze assembly mission critical testing does not provide prognostic information about electrical and electronic circuits and subtle causes of failure. Longer storage times and harsh conditions cause degradation at the component level. In order to calculate accrued damage due to storage and operational stresses, it is necessary to perform sample-based accelerated life testing after a certain time and estimate the remaining useful life of mission critical parts. Reliability studies of mechanical parts of such systems using nondestructive testing (NDT) have been performed, but a thorough investigation is missing with regards to the electronic parts. The objective of this study is to identify weak links and estimate the reliability and remaining useful life of electronic and detonating parts. Three critical components are identified in an electronic fuze circuit (1) a diode, (2) a capacitor, and (3) a squib or detonator. The accelerated test results reveal that after ten years of storage life, there is no significant degradation in active components while passive components need to be replaced. The squib has a remaining useful life (RUL) of more than ten years with reliability over 99%.

## 1. Introduction

Electronic fuze is a mission-critical part of one-shot systems that demands high storage and operational reliability. Reliability is defined as the time a system will perform its intended function without failure under stated operating conditions. One-shot systems such as air bags, firefighting equipment, initiating devices, electronic fuzes, and avionics assemblies of rockets are used once in their complete service life. During dormant life in storage, these devices undergo environmental stresses which cause degradation and can affect the operational reliability of the product. The service reliability requirement for such systems is very high due to the mission profile and application. Failure of any critical component due to storage fatigue and degradation can cause mission failure [1,2]. In order to make accurate estimations of remaining useful life (RUL) and reliability predictions of these systems and subsystems, it is necessary to examine the prognostic health of critical components. Accelerated life testing by simulating environmental conditions of long-term storage is used to quantify the health of critical components and their failures [3,4]. Repeated nondestructive testing (NDTs) and sample-based destructive testing over a minimum period of five years are essential to estimate the operational reliability of the mission-critical parts [5]. Functional testing with Boolean results of a pass-fail (Go/NoGo) does not provide any information about the degradation of components. Measured values of test results carry some degree of information about the degradation process and can be predicted by the drift in measured values towards failure. Environmental stresses applied to electronic modules cause degradation both in components and assembly (soldering), which leads to failure, especially at weaker links [6]. A one-shot system usually has long service and high-reliability requirements, and therefore failure-based removals by studying failure mechanisms help to achieve high reliability during the entire service life [7,8]. Reliability analysis and RUL estimation of small electronic explosive detonators (devices) also called EEDs is a critical issue in the field of safety and reliability due to the high risk in the case of failure. Reliability of miniature safety and arming devices (SADs) used in artillery fuzes is assured by conducting environmental tests under a variety of temperature, vibration, and impact conditions [9]. Researchers have worked on RUL and reliability estimations of the one-shot system using different techniques involving stress analysis, highly accelerated life testing (HALT) for failure analysis, and failure mode effect and criticality analysis (FMECA) to determine the reliability of subsystems and assemblies of the one-shot system. Martin [10] studied the monitoring and maintenance of the one-shot system. Martin further explored the relationship between destructive testing and fatigue degradation to establish repair criteria for the reliability assurance of a one-shot system. Wang et al. [11] studied the design of safety and arming design for a fuze at high speeds. Lall et al. [12,13] used a nondestructive method to evaluate the reliability of fuze electronics using finite element techniques to examine any damage to the electronic circuitry of a fuze. Yet the technique is unable to answer functional performance and degradation in electrical characteristics at the component level. Suhir [14] suggested that a probabilistic design for reliability (PDfR) was a better tool than HALT for the understanding of the operational reliability of new products for which no degradation data is available. Cheng et al. [15] and Cheng et al. [3] discussed the operational reliability of the one-shot system under normal operation and in a stressed thermal cycling environment. Sakamoto et al. [16] employed accelerated testing for the identification of failure modes for operational amplifier circuits. Xie et al. [17] worked on the design improvements and firing performance of silicon-based detonators. Chiodo and Lauria [18] discussed redundancies and the effect on the failure rate of industrial electronic applications. They discussed the relationship between failure rates and redundancies, for *k* out of *n* systems, and the effect on the overall reliability of the system. They also discussed the variation in failure rates due to intermittent properties of infant mortality and high failure rate which continuously decreases with time. After infant mortality, there exists a stable, low failure rate (operational life) and finally increasing failure rate towards the end of life for electronic products. Sharp et al. [19] discussed the design for reliability (DFR) for cluster munition fuze (CMF) with the reliability of more than 99%. They also explained the concept and introduction of redundancies in the case of a high-reliability application where human safety and operational reliability are required. Zhao and Yun [20] studied inspection intervals for the one-shot system. The development of the reliability block diagram and then identifying parallel and series systems contributing towards reliability can help in the overall system reliability estimation of the electronic module [21]. Wang et al. [22] measured and analyzed solid-state drives (SSD) reliability using accelerated testing. They compared the results and retention of properties both at normal and high temperatures. Data collection to estimate degradation is performed over a certain period, and the measurement equipment needs to be reliable for accurate data collection. Micro-electromechanical systems (MEMS) based technology is replacing conventional electronic detonators, but still, many systems are presently in storage [23].

Electronic fuzes, electronic explosive detonators (EEDs), semiconductor bridges (SCB), squibs, and detonators are a part of almost every artillery munition [24,25,26,27]. The devices are kept in storage for years before they need to be used [28]. The concept of interval inspection and RUL estimation is very common in the defense industry, and some time life extension of these assets is carried out based on prognostic health of the one-shot system. From the above literature and to the best of our knowledge, there exists a gap in the area of reliability estimation and RUL calculation for a one-shot system based on component degradation analysis due to storage stresses. In the literature, there is no significant work in the area of remaining useful life estimation using degradation analysis and accelerated life testing for failure analysis of electronic components [29,30,31]. In this research article, an electronic fuze is divided into three parts, the electronic part that provides the initial trigger, the chemical part that produces an explosion, and finally the mechanical part that provides arming and travel of the explosive train. This research article only elaborates the first two parts, while the reliability of the mechanical part is taken as unity. Accelerated life testing of critical electronic components is carried out to obtain degradation and failure data. Reliasoft ALTA-7 is used for degradation data analysis of electronic components with fail (F) or sustain (S) type data inputs, and Weibull analysis is performed to estimate standard and conditional reliability of the device under test (DUT). Grey forecasting models, GM (1,1) and discrete grey forecasting model, DGM (1,1), are used to estimate RUL using the degradation of the explosive part as it provides the best estimation under a limited data environment [32].

The outcome of this study is the identification of a weak link that affects reliability, and needs repair, replacement, or redesign to ensure overall system reliability and RUL. The main focus of this study is to determine the electronic module failure data and its reliability estimation using Reliasoft ALTA and Weibull. The grey forecasting model, GM (1,1), is used for the degradation analysis of squib decreasing pressure [33,34]. The results show that the capacitor is the weakest link, while all other parts have a reliability of more than 99% for a minimum RUL of five years.

The contents of this manuscript are as follows: Section 2 discusses accelerated life testing and Weibull analysis of degradation and failure data, Section 3 contains a brief explanation of the grey forecasting models, Section 4 describes the methodology of the research work, Section 5 is about the result and discussion of the degradation analysis, and Section 6 summarizes our conclusions.

## 2. Accelerated Life Testing and Weibull Analysis

**Figure 1:**
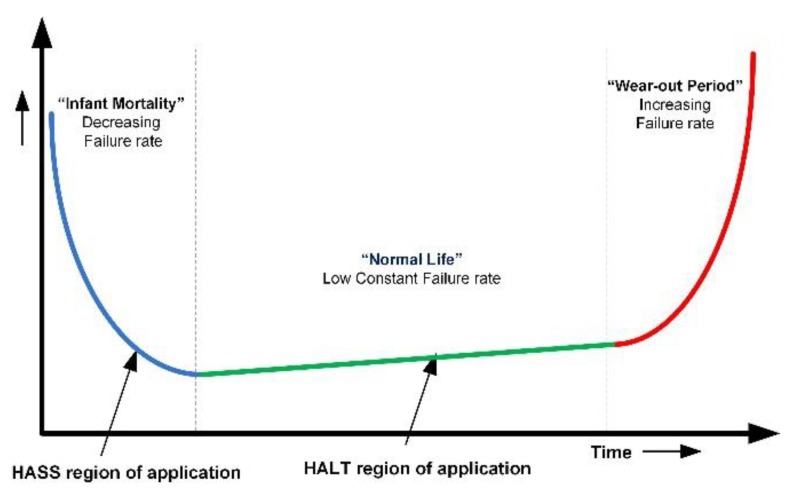
Bathtub curve for electronic products.

The concept of the famous bathtub curve provides an idea of life cycle of electronic assemblies as worst when new due to infant mortality and a high failure rate decreasing with time. Accelerated aging (AA) and highly accelerated stress screening (HASS) are used to precipitate failures due to infant mortality [35,36]. After the infant mortality period, electronic products have a small constant failure rate and, finally, the wear-out period is usually achieved by highly accelerated life testing (HALT), as shown in Figure 1.

An electronic fuze has three critical components that are affected by storage stresses and undergo degradation. They include a diode in the booster circuit, a capacitor as a source of energy for a squib or detonator, and a detonator or squib itself [37,38,39]. Due to their role in the successful operation of electronic fuzes, there RUL and reliability affect the overall life and reliability of the one-shot system. The following models are used to calculate testing time, considering the storage and operational stresses of temperature and humidity. Acceleration factor is used to calculate the HALT testing time at given stress values to acquire degradation or failure data [16].

During the storage of electronic explosive devices, two types of stresses are considered. One is thermal storage stress as storage temperature varies from location to location and the other is humidity which also varies with time and place of storage. To simulate these stresses, we use two models to calculate stress levels and test time to perform HALT. This is to simulate the stress models and to predict degradation in the electronic part of EED, and hence the RUL. The Arrhenius model temperature stress and Arrhenius relationship are given as [40]:

(1) $$R(T)=Ae^{{}^{\frac{-Ea}{kT}}}$$

where *R*(*T*) is reaction rate, *A* is nonthermal constant, *Ea* is the activation energy, k is the Boltzmann’s constant 8.6173303 × 10^−5^ eV/K, and *T* is the absolute temperature (Kelvin).

(2) $$\mathrm{Acceleration}\;\mathrm{Factor}=Af=\frac{TTFF}{TTFT}=e^{{}^{\frac{-Ea}{kT}\times (\frac{1}{T1}-\frac{1}{T2})}}$$

where *Af* is the acceleration factor, *TTFF* is time to failure in the field, *TTFT* is the time to failure in the test, *T*_1_ is the operating temperature also called low temperature, and *T*_2_ is the test temperature also called high temperature. In accelerated testing, test temperature is always higher than operating temperature. The Arrhenius exponential reliability function is given by:

(3) $$R(T,V)=e^{Ce^{\frac{-T}{\frac{B}{V}}}}$$

For the Arrhenius exponential model, the reliable life *t_R_* is given as:

(4) $$t_{R}=-Ce^{\frac{B}{V}}\ln[R(t_{R},V)$$

Combining the Arrhenius model with the Weibull distribution, we get:

(5) $$t_{R}=C\cdot e^{\frac{B}{V}}{\left\{-\ln[R(t_{R},V)]\right\}}^{\frac{1}{\beta }}$$

In the above equations, parameters B and C are called Arrhenius parameters also known as model parameters. In the linearized Arrhenius equation, ln(C) is the intercept of the line and B is the slope of the line. The Arrhenius parameter B is linked to activation energy $E_{a}$ and is given as $B=\frac{E_{a}}{K}$, where K is Boltzmann’s constant. Parameter *C* is linked with life L, stress V and B, and is given as $ln(L(V))=ln(C)+\frac{B}{V}$, where L(V) represent life L at stress level V.

During storage, one more stress along with temperature that causes degradation is humidity, given as:

(6) $$Af={\left(\frac{H_{Stress}}{H_{Use}}\right)}^{m}$$

The Arrhenius–Peck model is a combination of humidity and temperature stress to simulate the storage condition and is given as [41]:

(7) $$Af={\left(\frac{RH_{Stress}}{RH_{Use}}\right)}^{m}e^{{}^{\frac{-Ea}{kT}\times (\frac{1}{T1}-\frac{1}{T2})}}$$

The components are subjected to environmental stresses, and time and stress levels are calculated as per the acceleration factor. The failure or degradation data can be analyzed using Reliasoft Weibull++ and Reliasoft ALTA to estimate the degradation trends and reliability.

The Weibull probability density function (pdf) is given as:

(8) $$f\left(t\right)=\frac{\beta }{\eta }{\left(\frac{t-\gamma }{\eta }\right)}^{\beta -1}e^{-{\left(\frac{t-\gamma }{\eta }\right)}^{\beta }}$$

where *f*(*t*) ≥ 0, *η* is the scale parameter or the characteristic life (*η* > 0), *β* is the shape parameter (*β* > 0), and *γ* is the location parameter or the failure-free life with *t* ≥ *γ* (−∞ < *γ* < +∞). The Weibull cumulative density function is given as:

(9) $$F\left(t\right)=1-e^{-{\left(\frac{t-\gamma }{\eta }\right)}^{\beta }}$$

and reliability function is given as:

(10) $$R\left(t\right)=1-F(t)=e^{-{\left(\frac{t-\gamma }{\eta }\right)}^{\beta }}$$

In this paper, Equations (2), (6), and (7) are used to calculate the acceleration factor with operational and storage stresses, whereas, Equations (9) and (10) are used to calculate the pdf and the reliability of the critical components.

## 3. Grey Forecasting Models

The grey forecasting models are used to predict future values of the squib pressure to calculate the RUL of the detonating part of the fuze. The sample size is usually large to make a prediction and life estimation, as we need reliability of more than 99%, with a confidence level of 95%. The basic form of grey prediction model GM (1,1) is explained below [33,34]:

**Step 1** Sequence of original values

(11) $$x^{\left(0\right)}=\left\{x^{\left(0\right)}\left(1\right),x^{\left(0\right)}\left(2\right),\;x^{\left(0\right)}\left(3\right),\;\ldots ,x^{\left(0\right)}\left(k\right),\;\ldots ,x^{\left(0\right)}\left(m\right)\right\};$$

**Step 2** The accumulating generation operator (AGO) sequence is given as

(12) $$x^{\left(1\right)}=\left\{x^{\left(1\right)}\left(1\right),x^{\left(1\right)}\left(2\right),\;x^{\left(1\right)}\left(3\right),\ldots ,x^{\left(1\right)}\left(k\right),\;\ldots ,x^{\left(1\right)}\left(m\right)\right\}$$

where $x^{\left(1\right)}\left(k\right)=\overset{k}{\underset{i=1}{\sum }}x^{\left(0\right)}\left(i\right)$ and k=1,2,3, …,m;

**Step 3** The inverse accumulating generation operator (IAGO) sequence is given as

(13) $$\frac{d{\hat{x}}^{\left(1\right)}}{dt}+a{\hat{x}}^{\left(1\right)}=b$$

where ${\hat{x}}^{\left(1\right)}\left(1\right)\;\mathrm{is}\;\mathrm{predicted}\;\mathrm{value}\;\mathrm{of}\;x^{\left(0\right)}\left(1\right)$;

**Step 4** Predicted values value ${\hat{x}}^{\left(1\right)}$ is expressed as

(14) $${\hat{x}}^{\left(1\right)}≅Px^{\left(1\right)}\left(k\right)+\left(1-P\right)x^{\left(1\right)}\left(k+1\right)=z^{\left(1\right)}\left(k+1\right)$$

where *k*  = 1,2,3,….

Here *P* is a predictor parameter and traditionally its value is taken as 0.5 in the original model.

GM (1,1) is given as

(15) $$x^{\left(0\right)}\left(k\right)+az^{\left(1\right)}\left(k\right)=b$$

where k = 1,2,3,… .

Model parameters *a* and *b* in Equation (7) can be expressed as

(16) $$\left[\begin{array}{l} a\\ b \end{array}\right]={\left(B^{T}B\right)}^{-1}B^{T}Y_{N}$$

B and $Y_{N}$ in Equation (8) can be expressed in the following matrix form as

(17) $$B=[\begin{array}{l} -Z^{\left(1\right)}(2)\\ -Z^{\left(1\right)}(3)\\ \vdots \\ -Z^{\left(1\right)}(m) \end{array}\begin{array}{l} 1\\ 1\\ \vdots \\ 1 \end{array}]Y_{N}=\left[\begin{array}{l} x^{\left(0\right)}(2)\\ x^{\left(0\right)}(3)\\ \vdots \\ x^{\left(0\right)}(m) \end{array}\right]$$

**Step 5** The particular solution in the form of model parameters *a* and *b* as

(18) $${\hat{x}}^{\left(1\right)}\left(k+1\right)=\left(x^{\left(0\right)}\left(1\right)-\frac{b}{a}\right)e^{-ak}+\frac{b}{a}$$

where k=k=1,2, …, n−1,

Solving Equation (13), we get Equation (18), which is called time response, the corresponding predicted output is given by the following equation:

(19) $$\begin{array}{ll} {\hat{x}}^{\left(0\right)}x\left(k+1\right) & ={\hat{x}}^{\left(1\right)}x\left(k+1\right)-{\hat{x}}^{\left(1\right)}x\left(k\right)\\ & =\left(1-e^{a}\right)\left(x^{\left(0\right)}\left(1\right)-\frac{b}{a}\right)e^{-ak} \end{array}$$

where *k* =1,2,3,….

## 4. Methodology

**Figure 2:**
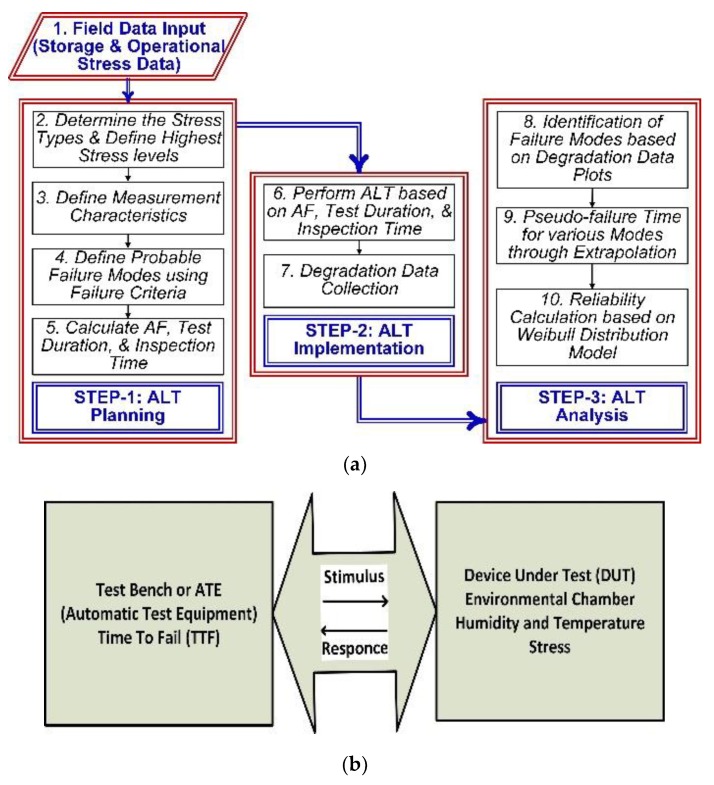
The proposed methodology for the remaining useful life (RUL) and reliability calculations. (**a**) Methodology and process flow diagram; (**b**) experiment procedure block diagram.

In this section, the method for calculating the test time of each component for an accelerated-aging equivalent to five years using Equations (2) to (7) is explained. Furthermore, the components or modules are subjected to different stress levels to obtain the degradation data as well. This degradation data is, then, used as an input to Weibull++ and ALTA-7 (Equations (8) to (10)) to estimate the failure and reliability of components. Weaker links in randomly selected twenty printed circuit boards (PCBs) of the electronic fuze are selected by design analysis and field failure data. Three components, the diode, the capacitor, and the squib are selected as the weaker links as these are responsible for providing the trigger current and energy to the squib which is necessary to perform the intended function. Using temperature and humidity models from Equations (4), (5) and (7), the acceleration factors and test time under stated conditions is calculated. The storage temperature is taken as 25 °C to 35 °C (*T*_1_) and the accelerated temperature as 100 °C to 125 °C (*T*_2_) for active components and 85 °C for the passive component. The relative humidity is taken from 40% to 85% and activation energy *Ea* = 0.7–0.9 eV. Using these values and acceleration factor *Af,* the test duration at designated stress levels is calculated for all three types of components. In order to calculate the reliable life for the next five years, we perform highly accelerated life testing (HALT) on 20 samples of each component. The HALT testing time or the accelerated aging time for capacitors result is 80 h and for diodes, it is 64 h. The flow diagram of the complete methodology ALT planning, implementation, and analysis is shown in Figure 2a and a block diagram of the test setup to acquire degradation or failure data is shown in Figure 2b.

**Figure 3:**
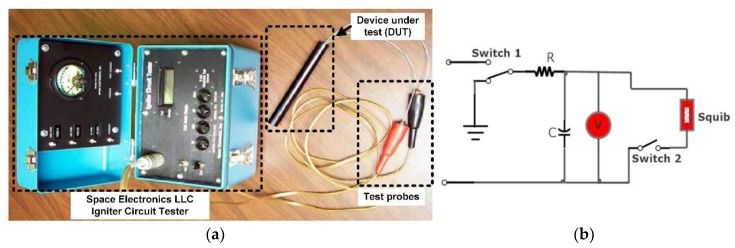
Squib electrical properties measurements. (**a**) Squib or ignitor tester; (**b**) squib or ignitor circuit.

As shown in Figure 2b, the environmental chamber is interfaced with the automatic test equipment (ATE) or measuring instrument and the results are tabulated after every four hours. In the case of failure of a component, the previous pass time is recorded as TTF (time to fail). During ALT, either the component fails (F) and we get time to failure (TTF) or the component is suspended (S) and the state end time is equivalent to TTF during the test. In data analysis using ALTA, it is necessary to mention the F or the S state along with TTF or state end time. The F and S values and corresponding time are calculated for both diode and capacitor as the input data for ALTA and Weibull analysis to calculate RUL and reliability using the above setup in Figure 2b. In Figure 3a, a squib tester is used to measure electrical characteristics of the squib. The performance of the detonating part depends upon both the electrical and explosive properties of the squib. A change in squib resistance and firing current can lead to failure. Once the squib is qualified for electrical characteristics, the chemical properties and explosion pressure provide the operational ability to perform its intended function. In Figure 3b, when Switch 2 is closed, it provides a discharging path to the capacitor through the squib resistance. The energy stored in the capacitor is dissipated in the squib causing it to produce an explosion. The health of the capacitor assures the strength of the explosion, which is required to initiate the explosive train as mentioned in [11], and therefore the capacitor is a critical component.

For the squib, the relative humidity (RH) is taken as 40% and 70%, *Ea* = 1.00704 eV, and the temperature as 25 °C (*T*_1_) and 70 °C (*T*_2_). Using Equation (7) the A*f* is calculated to be 916.31 and the test time for ten years of reliability as 94.29 h. For the squib testing, we normally measure firing current, no firing current, ohmic values, and finally firing power. Because the squib demands high reliability, i.e., around 99.5% with a confidence level of 95%, therefore, the sample size is usually large even with a zero failure rate. Typically, 600 samples are required with zero failure and for a single failure, the sample value rises to 950 in order to assure 99.5% reliability with a confidence level of 95%.

Firing power after qualification of electrical parameters for all squibs is measured at the end of accelerated aging for 2.5, 5, 7.5, and 10 years or at 23.5, 47, 70.5, and 94 h, respectively. The firing power decreases with time, but remains above some minimum threshold value which is 10 MPa. The decline in firing power is due to storage stresses, especially temperature and humidity. As the squib is a critical part and is vital in a successful mission completion, therefore, a larger sample size is subjected to destructive testing to measure firing pressure and reliability.

**Figure 4:**
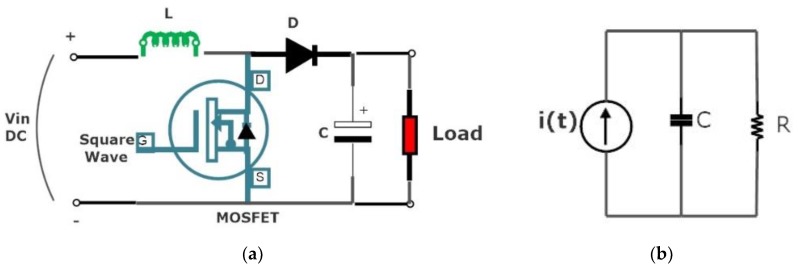
Squib energizing circuit. (**a**) Booster circuit for high current; (**b**) capacitor discharging circuit.

In order to provide the required charging current for the capacitor, we used a booster circuit that consisted of the MOSFET, diode, and bleeding resistor, as shown in Figure 4a,b. As the energy stored in a capacitor is given as energy stored in capacitor = $\frac{1}{2}CV^{2}$, therefore, capacitance and voltage developed across the capacitor are important parameters. This voltage can drop in the presence of leakage current of the capacitors and this will affect the firing current required to initiate an initial trigger for the explosive train through the squib resistance. Therefore, the decline in capacitance or the increasing trend of leakage current in long term storage application is critical for mission reliability.

**Figure 5:**
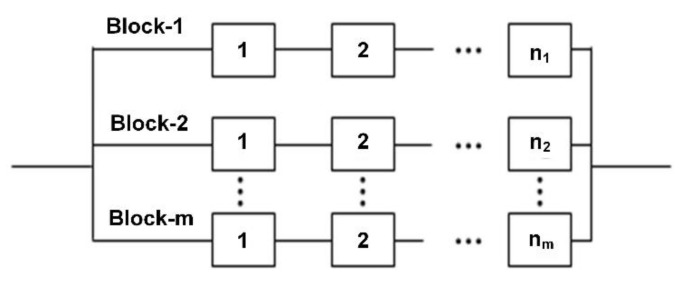
Parallel series system structure.

The failure of the diode is caused by diode forward current *I_r_* and *V_F_*, as the diode acts as protection in the booster circuit. Finally, after calculating individual reliabilities, we use series and parallel combinations of reliabilities to calculate the reliability of the electronic fuze, as shown in Figure 5. As all these parts are connected either in series or parallel combination, and therefore combined reliability is calculated based on series and parallel combination of the reliabilities of individual components.

## 5. Results and Discussion

After calculating stress levels (acceleration factor) and testing times for the weaker links of the electronic fuze, accelerated life testing is performed to obtain failure or degradation data. The analysis of failure or degradation data is performed using Reliasoft ALTA and Weibull to predict the failure of the component. The accelerated life test for the first weaker link capacitor is performed for a reliable life of ten years. The required testing conditions for 20 samples of capacitors for accelerated testing are: Temperature = 80 °C, relative Humidity = RH = 85%, and test time = 80 h. Degradation data for all the 20 capacitors are analyzed using ALTA to study the extent of degradation due to accelerated aging and the ability of the capacitor to withstand environmental stresses for a period of ten years. The degradation results show that all 20 capacitors are still in the qualified zone of their characteristic curves, but the trend is moving towards failure and the curve becomes steeper as we approach the time limit of 80 h. Humidity and high temperature negatively affect the dielectric strength causing an increase in leakage current and a decrease in charge µholding capacity or capacitance. An LCR meter (Agilent 4284A) interfaced with the environmental chamber was used for sequential testing of capacitors.

**Figure 6:**
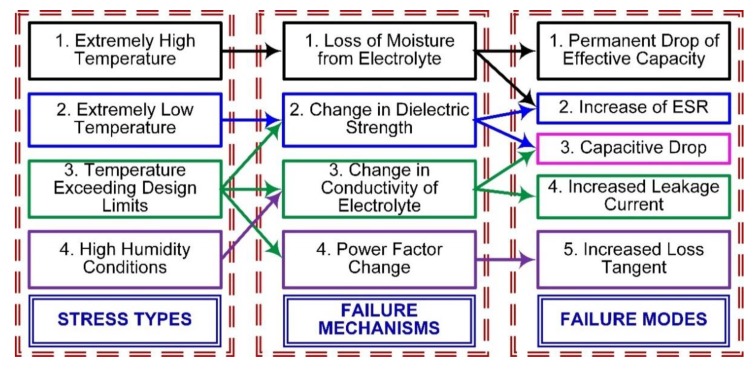
Stresses, failure mechanism, and failure modes in the capacitor.

Failure due to different stresses, the failure mechanism, and the failure modes are shown in Figure 6. The physics of failure in the different components help to identify the stresses and their impact on the life of the components. Knowledge of the failure mechanism helps to identify the impact of a stress environment on different parameters, and failure modes help to screen out the underperforming components. The failure mechanism and failure modes help to improve the operating conditions and protective measures, and therefore avoid failures. For active components such as the diode, transistors, and integrated circuits, the life span is large and failure is mostly linked with wear out, quality of material, and doping. For the power devices, failure is linked with heat dissipation and the load attached. For passive components such as capacitors, the effect of environmental stresses is more rapid. Therefore, for a product consisting of different types of components, it is necessary to identify weaker links and estimate RUL and reliability. Knowledge of stresses, the failure mechanism, and failure modes helps with the selection of a capacitor for operation in a stressed environment and also to identify critical parameters during accelerated aging. The failure of a fuze can be due to mechanical failure or electrical failure, electrical failure due to components is discussed here. Another failure can be due to solder joints and Muhammad et al. [6] discussed the effect of environmental stresses on solder joints. The analysis of degradation or failure data received from field failure or acquired by HALT helps in forecasting reliability and RUL.

**Figure 7:**
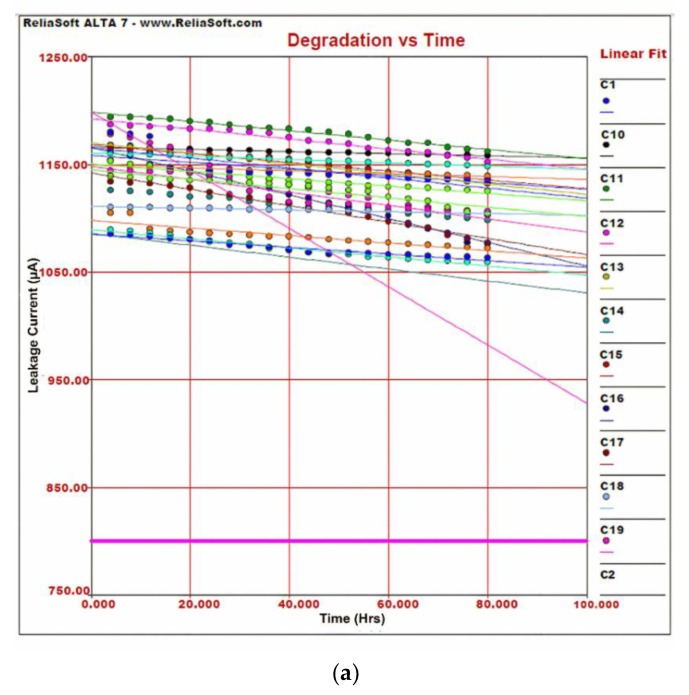

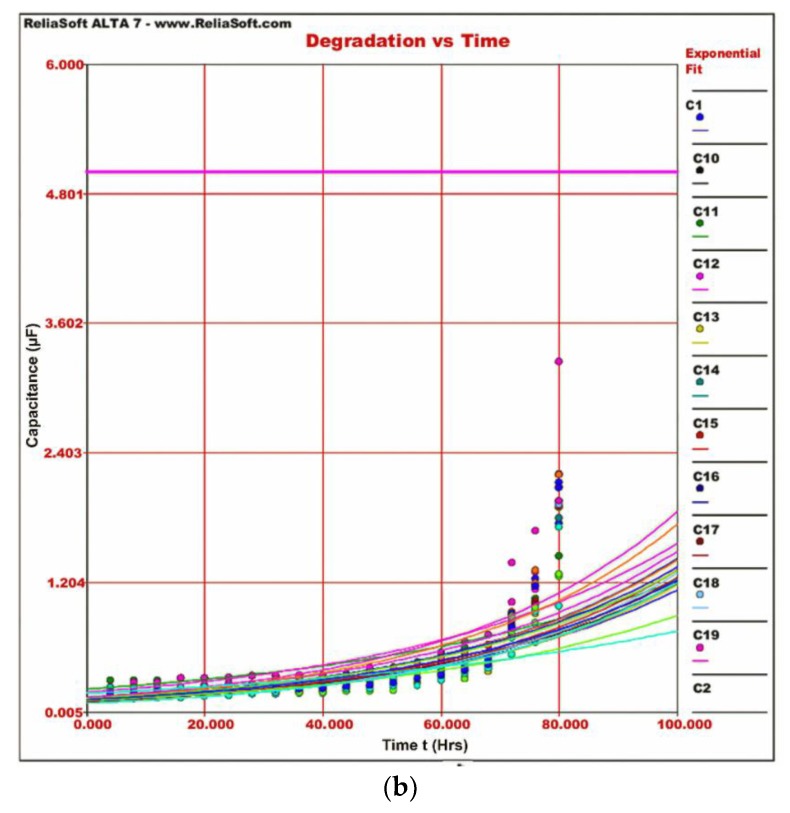
Capacitor degradation vs. time. (**a**) Leakage current vs. time; (**b**) capacitance vs. time.

The decrease in capacitance and increase in the leakage current of the capacitor with time are shown in Figure 7a,b. Figure 7a,b also shows that, at time *T* = 80 h, all the important characteristics of capacitors are well within range but show a degrading trend. One capacitor shows an abnormal trend and is heading towards failure. This shows that capacitors need to be replaced in order to achieve high reliability. The tendency of failures can be observed if we extrapolate the curves in Figure 7a beyond 80 h. The trend shows that humidity and temperature stresses in accelerated aging have damaged the core parameters of capacitors and the reliability of both component and product is compromised. The results help in the identification of the critical component that can affect the overall reliability of the product and needs replacement every five years instead of ten years. Over time, the capacitor loses its capacitance due to degradation in dielectric strength. Because the trigger current for initial explosion is provided by the energy stored in the capacitor, therefore capacitance becomes a critical parameter for mission success. From the graph in Figure 7a,b we observe that due to degradation in parameters, a few more capacitors can suddenly lose their characteristic during the next five years of storage. The cost of replacing a component is less than the cost of failure and high reliability of a product demands even higher component reliability. To achieve this, early removal of a capacitor is recommended. When the capacitor is replaced, in the overall reliability calculation, the value of the RC is taken as 0.995, which is the same as taken at the start of the life cycle. As storage has a negative influence on capacitance, therefore, electrolytic capacitors must be scrutinized for the date of manufacturing before use in critical applications.

**Figure 8:**
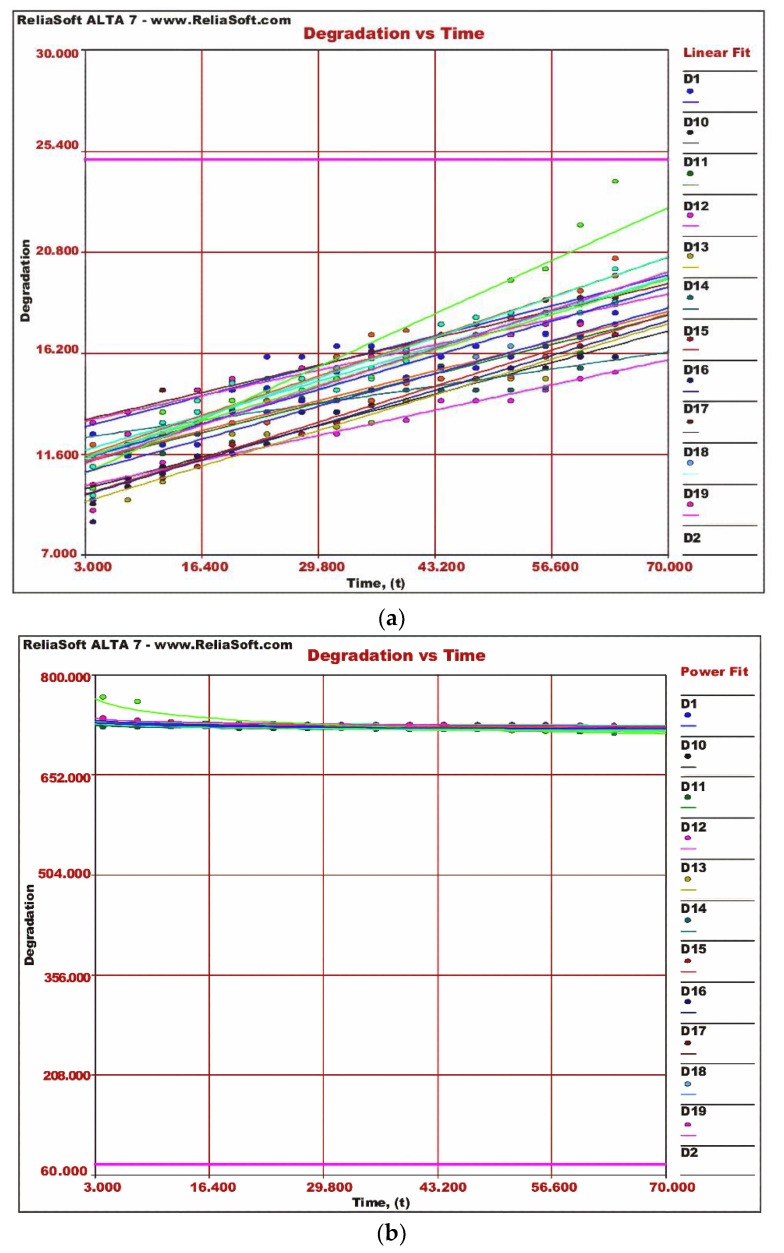
Degradation vs. time. (**a**) Diode *I_r_* vs. time; (**b**) diode *V_F_* vs. time.

The second weaker link is the diode in the electronic fuze, again twenty diodes are subjected to accelerated life testing at higher degrees of temperature. This helps to obtain the degradation data and to study the effect of thermal stresses on different characteristics of diode and failure modes triggered by these thermal stresses. The component datasheet, storage, and operational conditions provide useful information about stress levels, and hence the calculation of the acceleration factor. The acceleration factor, due to high-temperature stresses, is calculated by using the Arrhenius relation. The values obtained for forward voltage (*V_F_*) and leakage current (*I_r_*) at high temperatures after every four hours are plotted against time. A Tektronix Curve Tracer 370B is used for testing the diodes. Figure 8a,b shows that the forward voltage (*V_F_*) remains constant with time and is not affected by thermal stress. Contrary to this, the reverse current *I_r_* increases with time under thermal stresses which indicates that, at high temperatures, minority carriers’ increase.

Figure 8a,b shows that values of critical parameters for diodes do not exceed the threshold values during accelerated testing and are well within limits. It means the diode will not affect the overall reliability of the electronic fuze for the next ten years.

**Figure 9:**
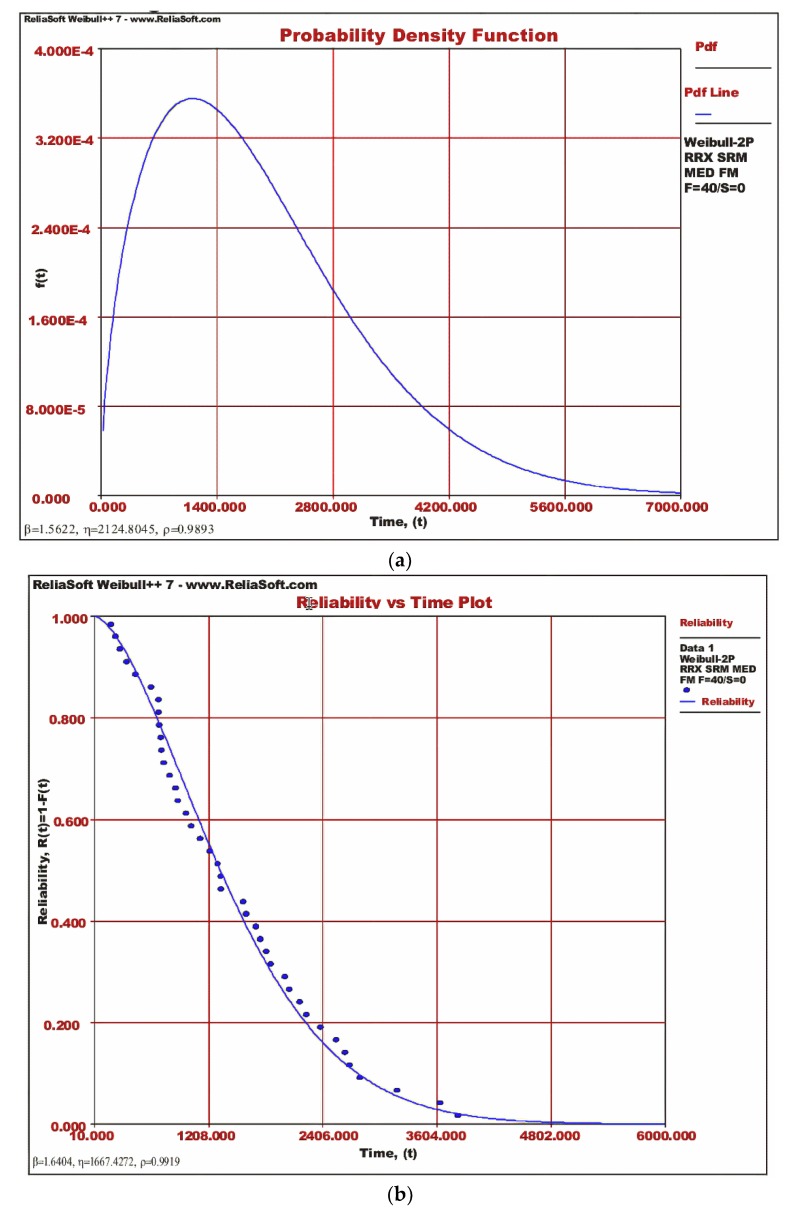
Reliability of diode (**a**) pdf plot for diode degradation and (**b**) reliability plot for the diode.

Finally, we use Weibull analysis to estimate standard and conditional reliability for the diode, as the capacitor needs to be replaced with a new one. Probability distribution function (pdf) plot and reliability plot for diode using Reliasoft Weibull function are shown in Figure 9a,b, respectively.

The conditional and standard reliability results from the probability distribution function are as follows:

- Standard reliability 0.99;
- Conditional reliability 0.989.

It shows that out of two critical components, one of the components (diode) holds its performance characteristics and confirms ten years of reliable life while the capacitor needs to be replaced every five years.

In order to calculate the reliability and life of the electronic fuze, we need to calculate the reliability of both electronic and explosive parts. We perform accelerated life testing to evaluate the reliability and remaining life of the electronic components. The storage conditions, especially humidity, affect the performance of explosive parts by reducing the exploding pressure necessary for the reliable operation of the electronic fuze. As discussed in Section 4, a lot of samples are required to calculate the reliability of the explosive parts of an EED. Electrical characteristics of a squib need to be evaluated and, then, the exploding pressures need to be measured. The electrical characteristics of the filament or squib are as follows:

- Bridge resistance < 1.5 Ω, firing current > 1.5 A, Isolation resistance > 20 M Ω at 500 V;
- The firing qualified pressure range for the squib is 10 to 20 MPa.

The pressure measurement of samples is taken at different time intervals (years) T0, T2.5, T5, T7.5, and T10, by actually firing the samples. The pressure drop at T10 is well above the threshold value of 10 MPa. The grey forecasting model GM (1,1) provides an accurate prediction when the input data is limited. Here we have only four average values of detonating samples collected after 2.5 years. Equation (19) is used to calculate a prediction of future values in GM (1,1). Using the grey forecasting model GM (1,1) the values show that the total life of the detonator is more than 20 years, with the remaining useful life of more than 10 years as shown in Table 1.

The detonator has a reliable life for the next ten years or more. Using a series combination of electronic parts used as a current source or power source and explosion part, the reliability is the product of both parts. Therefore, the overall reliability of the electronic fuze is the product of individual reliabilities and is given as:

(20) $$R_{system(ElectronicFuse)}=R_{D}\times R_{S}\times \left(R_{M}-R_{M}\times R_{C}-R_{C}\right)$$

where Rsystem (electronic fuze) = 0.99 or 99% is the system reliability, *R_Diode_* = *R_D_* = 0.995, *R_MOSFET_* = *R_M_* = 0.995, *R_Capacitor_* = *R_C_* = 0.995, and *R_Squib_* = *R_S_* = 0.995. Alternatively,

(21) $$R_{system(ElectronicFuse)}=R_{D}\times R_{S}\times \left(R_{M}-R_{M}\times R_{C}-R_{C}\right).$$

Reliability electronic fuze = Reliability of electronics components × reliability squib = 0.995 × 0.995 = 0.99 or 99%.

If the sample size is increased, reliability is above 99%. Nevertheless, in the presence of limited data and samples, the estimated reliability can be considered as acceptable.

## 6. Conclusions

In this study, the reliability of the electronic parts of a fuze (one-short system) is calculated. An electronic fuze is a small one-shot device that is mission critical and consists of two parts. The electronic part provides the trigger current and has three critical components. The reliability of all other parts, including biasing resistors and the PCB, is taken as unity, as they are either not critical or not considered. The RUL of the PCB and solder joints is calculated separately. The second part is the explosive part and the squib which provides initial flame and, then, an explosion for successful completion of the mission. The overall results are in close agreement with the required reliability of 99%. The reliability of electronic fuze and squib, to the best of our knowledge, has not been studied at the component level. Normal studies used the Military Handbook 217 values for allocation or estimation of reliabilities. However, in this research work, actual values of constituent parts have been evaluated to calculate component and system reliability. Reliasoft was used to perform the accelerated life testing and degradation analysis. The Reliasoft Weibull distribution is an excellent tool for reliability calculations using degradation and field failure data. The complex system is further divided into smaller systems to calculate the reliability of smaller components which are further combined using a series and parallel combination to calculate the system’s reliability. This study also revealed that a design improvement is required, which replaces energy storing capacitors with others having long storage characteristics. In the future, the reliability of a complex system consisting of multiple subsystems could be calculated, where required, at the subsystem level, and later combined to calculate the system level reliability.

## Figures and Tables

**Table 1:** Grey forecasting values of detonators using GM (1,1).

Time Interval	Time (Years)	Actual Data	GM (1,1) Forecasted Data
T1	2.50	18.85	18.53
T2	5.00	17.35	17.20
T3	7.50	15.65	15.95
T4	10.0	14.98	14.80
T5	12.5	-	13.74
T6	15.0	-	12.75
T7	17.5	-	11.83
T8	20.0	-	10.97
T9	22.5	-	10.18
T10	25.0	-	09.45
	**Average Relative Error**	-	**0.06%**
